# Behavioral Health Screening in Geriatric Primary Care in the Deep South

**DOI:** 10.1093/geroni/igaa057.3140

**Published:** 2020-12-16

**Authors:** Rebecca Allen, Anne Halli-Tierney, Dana Carroll, Amy Albright, Deanna Dragan, Gregg Bell, Brian Cox

**Affiliations:** 1 Alabama Research Institute on Aging, Tuscaloosa, Alabama, United States; 2 The University of Alabama, Tuscaloosa, Alabama, United States; 3 Auburn University Harrison School of Pharmacy, Tuscaloosa, Alabama, United States; 4 University of Alabama, Northport, Alabama, United States; 5 The University of Alabama College of Community Health Sciences, Tuscaloosa, Alabama, United States

## Abstract

Behavioral health screening by interprofessional teams practicing in outpatient geriatric primary care improves identification of patient cognitive functioning and emotional needs. On average, geriatrics clinic patients who consented to participate in research (N = 209; 74% women; 16.6% African American) were 76.7 years old. Patients had an average of 5.83 medical diagnoses. Only 26.2% of patients had scores indicating cognitive functioning within normal limits; 32.6% had scores indicative of mild neurocognitive disorder, and 41.2% had scores indicative of dementia at their baseline visit. Over 30% of patients reported clinically significant levels of depression or anxiety, and 16.5% of patients reported at least one indicator of hazardous alcohol use. Five-year longitudinal data analysis reveals multiple patient profiles. Behavioral health screening in primary geriatrics clinic care may help identify patient cognitive and emotional needs across time. Part of a symposium sponsored by the Mental Health Practice and Aging Interest Group.

